# Deep Sequencing of Influenza A Virus from a Human Challenge Study Reveals a Selective Bottleneck and Only Limited Intrahost Genetic Diversification

**DOI:** 10.1128/JVI.01657-16

**Published:** 2016-11-28

**Authors:** Ashley Sobel Leonard, Micah T. McClain, Gavin J. D. Smith, David E. Wentworth, Rebecca A. Halpin, Xudong Lin, Amy Ransier, Timothy B. Stockwell, Suman R. Das, Anthony S. Gilbert, Robert Lambkin-Williams, Geoffrey S. Ginsburg, Christopher W. Woods, Katia Koelle

**Affiliations:** a Department of Biology, Duke University, Durham, North Carolina, USA; b Duke Center for Applied Genomics and Precision Medicine, Durham, North Carolina, USA; c Laboratory of Virus Evolution, Emerging Infectious Diseases, Duke-NUS Medical School, Singapore; d J. Craig Venter Institute, Rockville, Maryland, USA; e hVIVO, London, United Kingdom; Wake Forest University

## Abstract

Knowledge of influenza virus evolution at the point of transmission and at the intrahost level remains limited, particularly for human hosts. Here, we analyze a unique viral data set of next-generation sequencing (NGS) samples generated from a human influenza challenge study wherein 17 healthy subjects were inoculated with cell- and egg-passaged virus. Nasal wash samples collected from 7 of these subjects were successfully deep sequenced. From these, we characterized changes in the subjects' viral populations during infection and identified differences between the virus in these samples and the viral stock used to inoculate the subjects. We first calculated pairwise genetic distances between the subjects' nasal wash samples, the viral stock, and the influenza virus A/Wisconsin/67/2005 (H3N2) reference strain used to generate the stock virus. These distances revealed that considerable viral evolution occurred at various points in the human challenge study. Further quantitative analyses indicated that (i) the viral stock contained genetic variants that originated and likely were selected for during the passaging process, (ii) direct intranasal inoculation with the viral stock resulted in a selective bottleneck that reduced nonsynonymous genetic diversity in the viral hemagglutinin and nucleoprotein, and (iii) intrahost viral evolution continued over the course of infection. These intrahost evolutionary dynamics were dominated by purifying selection. Our findings indicate that rapid viral evolution can occur during acute influenza infection in otherwise healthy human hosts when the founding population size of the virus is large, as is the case with direct intranasal inoculation.

**IMPORTANCE** Influenza viruses circulating among humans are known to rapidly evolve over time. However, little is known about how influenza virus evolves across single transmission events and over the course of a single infection. To address these issues, we analyze influenza virus sequences from a human challenge experiment that initiated infection with a cell- and egg-passaged viral stock, which appeared to have adapted during its preparation. We find that the subjects' viral populations differ genetically from the viral stock, with subjects' viral populations having lower representation of the amino-acid-changing variants that arose during viral preparation. We also find that most of the viral evolution occurring over single infections is characterized by further decreases in the frequencies of these amino-acid-changing variants and that only limited intrahost genetic diversification through new mutations is apparent. Our findings indicate that influenza virus populations can undergo rapid genetic changes during acute human infections.

## INTRODUCTION

Advances in sequencing technologies have allowed researchers to observe, in unprecedented detail, the genetics of viral infections. In the case of infections caused by rapidly evolving RNA viruses, genetic sequence data have shown that viral pathogen populations within hosts frequently exist as complex populations of many genetically related virions (1, 2). Studies based on the analysis of next-generation sequencing (NGS) data have, in particular, shown that these populations can be highly diverse (3–5) and that they may evolve over the time course of a single infection (5–8). A number of studies have specifically focused on characterizing the evolutionary dynamics of influenza A virus (IAV), an important RNA virus with a broad host range and cross-species potential. These studies include ones that characterize IAV evolution across transmission events in attempts to determine the size and type of transmission bottleneck (9–19). While some of these studies have focused on the evolutionary dynamics of well-adapted viruses already circulating in these or similar host populations (9–16), others have instead probed the evolutionary dynamics of maladapted viruses in new host populations to determine the adaptive potential of IAV following cross-species viral spillover (17–19).

The studies that have considered IAVs that are well adapted to their hosts have generally shown that viral populations in recipient hosts are genetically similar to the ones in donor hosts and that they frequently harbor variants that were present at low frequencies in donor hosts (11–13, 16). These findings indicate that the between-host transmission dynamics of well-adapted IAVs are characterized by loose bottlenecks. The exact size of the bottleneck, however, depends on the route of transmission, as demonstrated in a recent small-mammal study using barcoded virus (15). Beyond viral evolution during the transmission process, these studies have further indicated that IAV genetic diversity can change over the course of acute infection through either genetic drift or natural selection (9, 10, 13). However, a general trend of intrahost viral diversification is not apparent in these studies (9, 10, 13).

Distinct from these studies, other studies have specifically focused on characterizing the evolutionary dynamics of viral populations during the process of host adaptation, where the virus is to some degree maladapted to the target host. The best known of these are the gain-of-function studies involving avian influenza virus adaptation to mammalian hosts via experimental infection of ferrets with avian influenza H5N1 (18, 20, 21), H1N1 (17, 22), or H7N9 (19) virus. These studies have generally shown that viral populations in recipient hosts differ from the ones in donor hosts, although they again can contain variants that are present at low frequencies in donor hosts (18). This finding suggests that the between-host transmission dynamics of less well adapted IAVs are instead better characterized by narrow, selective bottlenecks (17, 18), although the size and type of the bottleneck will be affected by the degree of viral maladaptation to the host (17). These selective bottlenecks appear to result mainly from strong selection on the hemagglutinin (HA) gene segment of the virus (18, 19). A subset of these studies have further shown that, following the profound reduction in viral genetic diversity during the process of transmission, intrahost IAV populations have the potential to diversify genetically over the course of acute infection (17, 18), although IAV genetic diversification is not apparent in all studies (19).

Here, we analyze a unique data set generated from a human challenge study in which subjects were experimentally inoculated with cell- and egg-passaged IAV. Both the viral stock used to inoculate the subjects and serial nasal wash samples collected from the subjects were deep sequenced. We first find that the viral stock had accumulated genetic variation during the passaging process, with some variants reaching high frequencies, presumably due to positive selection acting on these variants during the viral preparation process. We further find that IAV populations experienced a narrow, selective bottleneck following subject inoculation, similar to what was found in experimental studies with IAVs that were not well adapted to their target hosts (18, 19). In our case, however, and in contrast to some of these studies, we do not find evidence for extensive intrahost viral diversification following infection. Instead, we find evidence for continued purifying selection and only limited evolution via *de novo* mutation, more similar to what was found in studies with well-adapted IAVs.

## MATERIALS AND METHODS

### Approvals from IRBs.

The human influenza challenge study was conducted in 2008, with detailed descriptions of the study provided in references 23, 24, 25, 26, 27, and 28. The protocols used for the influenza challenge study were approved by the following institutional review boards (IRBs): the IRB of University Medical Center (Durham, NC), the SSC-SD IRB of the U.S. Department of Defense (Washington, DC), the East London and City Research Ethics Committee 1 (London, United Kingdom), and the Independent Western Institutional Review Board (Olympia, WA). As per standard IRB protocol, written informed consent was obtained from each participant in this study. All procedures in this study were in accordance with the Declaration of Helsinki.

### Subject enrollment and challenge study protocol.

The original purpose of this human challenge study was to develop an “acute respiratory viral signature,” defined as a set of changes in early host gene expression patterns occurring during the presymptomatic, postinoculation phase of infection that would be capable of identifying those individuals who would go on to later develop symptomatic influenza infection (27). To maximize infection rates, several subject exclusion criteria were set prior to participant enrollment. These exclusion criteria were influenza-like illness during the 45 days preceding the study, positive HA inhibition (HAI) titers to the challenge virus, and vaccination with a seasonal influenza vaccine within the preceding 3 years. A total of 17 participants were enrolled in this study, and based on the exclusion criteria, these participants were thus effectively immunologically naive to the challenge virus. These subjects were each intranasally inoculated with the challenge virus at doses ranging from 3.08 to 6.41 log_10_ 50% tissue culture infective doses (TCID_50_)/ml (25). Following inoculation, subjects were each monitored for a period of 7 days. All subjects in the study received oseltamivir treatment 5 days postinoculation. Of note, no association between the inoculum dose and the outcome of infection (presence/absence of infection, as determined by detection of virus in samples; symptomatic/asymptomatic infection in the case of virus detection) was detected within this challenge cohort (24). In the subjects who became infected (*n* = 9), no association between inoculum dose and the extent of viral shedding was found (24).

### Production of challenge virus.

**FIG 1:**
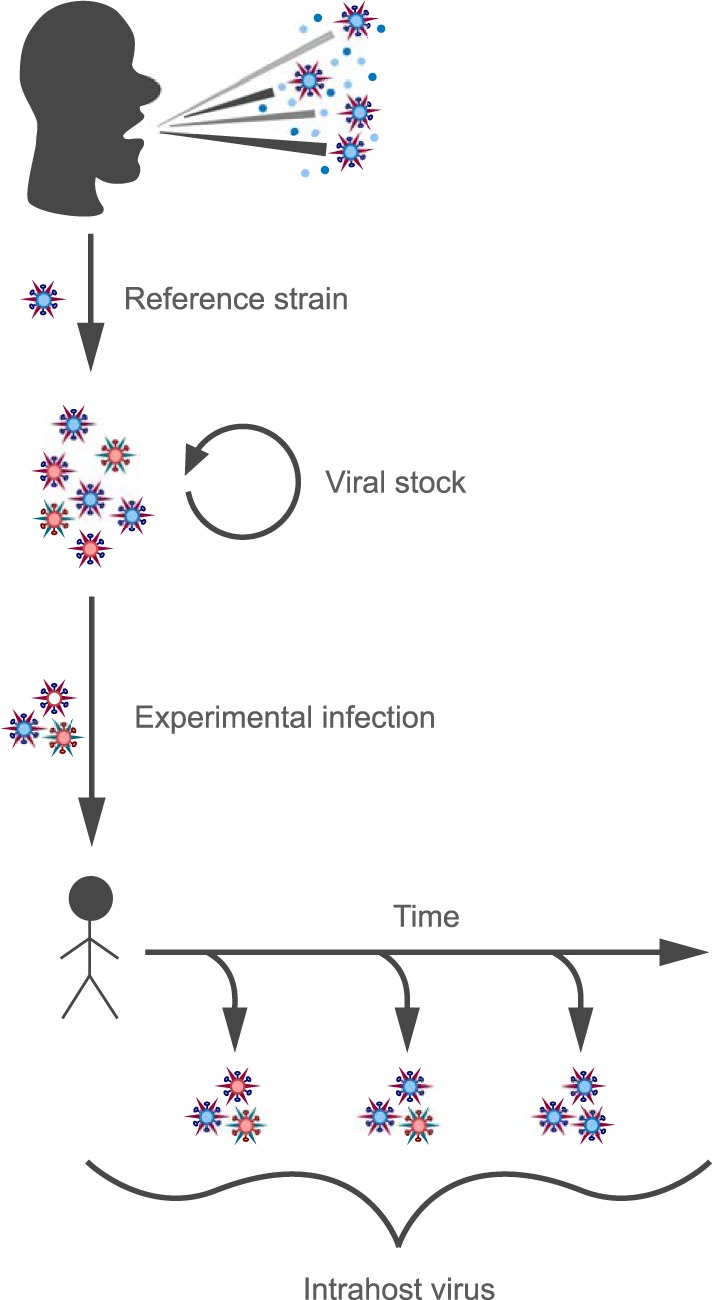
Schematic showing the relationship between the viral reference strain, the viral stock, and the nasal wash samples analyzed in this study. The viral reference strain A/Wisconsin/67/2005 (H3N2) was derived from a human clinical sample. This virus was passaged in avian primary chicken kidney cells, embryonated chicken eggs, and GMP Vero cells to generate the viral stock used to infect the challenge study subjects. Nasal wash samples were taken daily from each of the 17 subjects for 7 days following inoculation. Viral stock and nasal wash samples were subjected to NGS and analyzed.

The virus used for experimental challenge was produced under current good manufacturing practices (cGMP) at Baxter Bioscience (Vienna, Austria). The original virus was obtained from a human isolate of A/Wisconsin/67/2005 (H3N2) (GenBank accession numbers CY114381 to CY114388). We here refer to this human isolate as the “reference strain.” This virus was passaged 3 times in avian primary chicken kidney (CK) cells, 4 times in embryonated chicken eggs, and then twice in GMP Vero cells to generate the viral stock used to inoculate the challenge study subjects (Fig. 1).

### Virus sample isolation and next-generation sequencing protocol.

Nasal wash samples were obtained daily from each of the 17 subjects over the 7-day period of postinoculation monitoring. Each of these nasal wash samples was tested for virus using quantitative culture. Samples containing virus (a total of 26 samples of the 119 samples obtained) were sequenced at the J. Craig Venter Institute (JCVI) using an influenza NGS pipeline. Briefly, the NIH/NIAID-sponsored influenza NGS pipeline at JCVI uses a multisegment reverse transcription (RT)-PCR enrichment to amplify IAV's eight gene segments directly from the viral RNA extracted from the swab specimens (29, 30). Sequencing was performed using the Illumina HiSeq 2000 platform. In all, 12 of the 26 virus-containing nasal wash samples were successfully sequenced. Table 1 lists the subjects and sampling times corresponding to these 12 samples. Of the 7 subjects with at least one sequenced sample, 3 had a single sequenced sample, 3 had two sequenced samples, and 1 had three sequenced samples. Along with these nasal wash samples, the viral stock was deep sequenced using the same pipeline.

**TABLE 1:** Characteristics of successfully sequenced nasal wash samples by subject and sample day

Subject	Inoculum dose [log_10_ (TCID_50_/ml)]	Sample day	Quantity of virus in sample [log_10_ (TCID_50_/ml)]
S1	6.41	1	4.25
		2	4.25
S6	5.25	1	3.75
		3	3.25
S8	5.25	2	4.75
S10	4.41	3	3.75
S12	4.41	2	5.01
		3	5.01
		6	2.75
S13	3.08	2	3.51
		3	5.5
S15	3.08	4	3.75

### Sequence processing and variant identification.

Premapping quality control of the viral sequences involved removal of sequencing primers, followed by trimming of both the 5′ and 3′ ends of the short reads to remove regions where the probability of error was ≥0.01 per nucleotide, using Geneious Pro 8.0 (Biomatters Ltd., Auckland, New Zealand). Sequences <35 nucleotides in length following trimming were removed. The remaining sequences were assembled to the reference strain using the Burrows-Wheeler aligner (BWA) (31). Postmapping quality control of the sequences involved removal of PCR duplicates using Samtools “rmdup” (32), removal of short reads that contained insertions and deletions (33), and removal of short reads that were automatically trimmed by the assembler (“clipped ends”) (33). Following postmapping quality control, the assemblies were probabilistically realigned to correct mapping errors and then analyzed to detect variants using LoFreq (34). Based on a recent study of IAV genetic diversity (35), we selected the following criteria for calling single nucleotide polymorphisms (SNPs): Phred score of ≥35, mapping quality of ≥30, and *P* value of <0.01 (35). Based on the known error rate of the Illumina sequencing platform (36, 37) and the stringency of these variant call criteria, we used a minimum allele frequency cutoff of 1% to identify variants. Each of the identified variants was visually inspected and excluded if the variant was supported by <10 reads (38) or if the average read position of the variant occurred outside the middle 50% of the short read (35). We made one exception to these variant processing protocols. In one sample (subject S6 on day 3), the mean read position for T1680C on the PA gene segment was 24%, which fell slightly outside the middle 50% window. However, because this variant was present at high frequency in this sample (41%) and was detected in multiple other samples, we called it as a variant in this sample. Finally, nucleotide read numbers for each site at which an SNP was called were determined using Samtools “mpileup” (32) and VarScan “readcounts” (39, 40).

Note that the minimum allele frequency cutoff value chosen affects which variants are identified in a sample and, thus, could potentially affect the results of an analysis. To ascertain the sensitivity of our results to our chosen frequency cutoff of 1%, we repeated all analyses described below using a highly conservative frequency cutoff of 3%. There were neither qualitative differences nor quantitatively significant differences in our results when using a 3% frequency cutoff from when we used a 1% frequency cutoff. This shows that our results are robust to the exact frequency cutoff used.

### Pairwise genetic distance calculations.

Mean pairwise genetic distances between viral populations were calculated using the basic Euclidean distance metric, otherwise known as the L^2^ distance. The Euclidean distance between viral populations *x* and *y* at site *i* is given by

$$D_{i}=\sqrt{\sum_{j=A,T,G,C}{\left(f_{x,i,j}-f_{y,i,j}\right)}^{2}}$$

where *f_x_*_,*i*,*j*_ denotes the frequency of nucleotide *j* at site *i* in viral population *x* and, similarly, *f_y_*_,*i*,*j*_ denotes the frequency of nucleotide *j* at site *i* in viral population *y*. To limit the effects of background noise characterizing NGS data, we assumed that non-SNP sites were genetically monomorphic. That is, at a non-SNP site *i*, in population *x*, we let *f_x_*_,*i*,*j*_ be equal to 0 for three of the four nucleotides and let *f_x_*_,*i*,*j*_ be equal to 1 for the remaining nucleotide. Sites that had an identified SNP in one or more of the samples (including the viral stock) were considered polymorphic, with *f_x_*_,*i*,*j*_ ≥0 for each of the four nucleotides in each population *x* considered. When calculating *D_i_* between a viral population and the reference strain, we set the frequency of the observed nucleotide in the reference strain to 1 and the frequencies of the unobserved nucleotides in the reference strain to 0. For a focal gene segment, the mean genetic distance between viral populations *x* and *y* was calculated by averaging site-specific distances (*D_i_*) across the segment.

### Genetic diversity calculations.

Nucleotide diversity (π) was calculated as described in references 18 and 41 for the viral stock and the nasal wash samples. Briefly, nucleotide diversity at a site is calculated as the mean number of nucleotide differences between all pairs of sequences within a sample at the target site, mathematically given by

$$\sum_{i<j}\frac{d_{ij}}{(n^{2}-n)/2}$$

where *n* is the sample's coverage at the target site and *i* and *j* are reads spanning the target site. This diversity is then averaged across a gene segment to obtain π. As in references 17 and 18, we calculate both synonymous nucleotide diversity (π*_S_*) and nonsynonymous nucleotide diversity (π*_N_*), defined, respectively, as the mean number of synonymous substitutions per synonymous site and the mean number of nonsynonymous substitutions per nonsynonymous sites across a focal gene segment. The numbers of synonymous and nonsynonymous sites were estimated as described in reference 42. As in the pairwise distance calculations, we assumed that all non-SNP sites were genetically monomorphic and that only sites with identified SNPs in one or more samples were polymorphic.

### Accession number(s).

The assembled viral sequences can be accessed through the Sequence Read Archive (SRA) database (study SRP091397; accession numbers SRR4416113 to SRR4416125).

## RESULTS

### Quality and depth of NGS samples.

**FIG 2:**
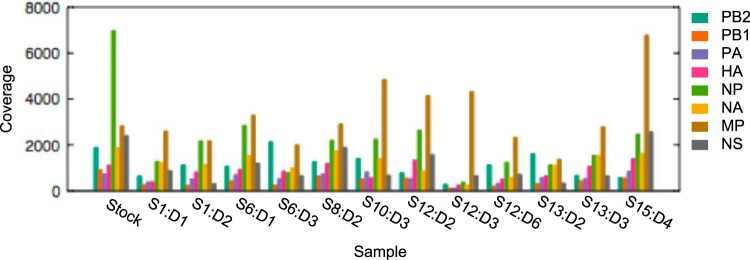
Mean read coverage for the viral stock and the 12 successfully sequenced nasal wash samples, by gene segment. Nasal wash samples are labeled with S*X*:D*Y*, where *X* is the subject number and *Y* is the postinoculation sampling day. Coverage levels were calculated following pre- and postmapping quality control.

The processed reads had an average length of 69.7 (±6.3) nucleotides and an average quality score of 34.4 (±5.0). The average per-site, per-sample coverage was 1,032 reads. Figure 2 shows mean read coverage levels by gene segment and sample, including the viral stock. The nasal wash sample from subject 12 on day 3 had particularly low coverage for all gene segments besides MP and NS.

### Extensive viral evolution occurred in the human challenge study.

**FIG 3:**
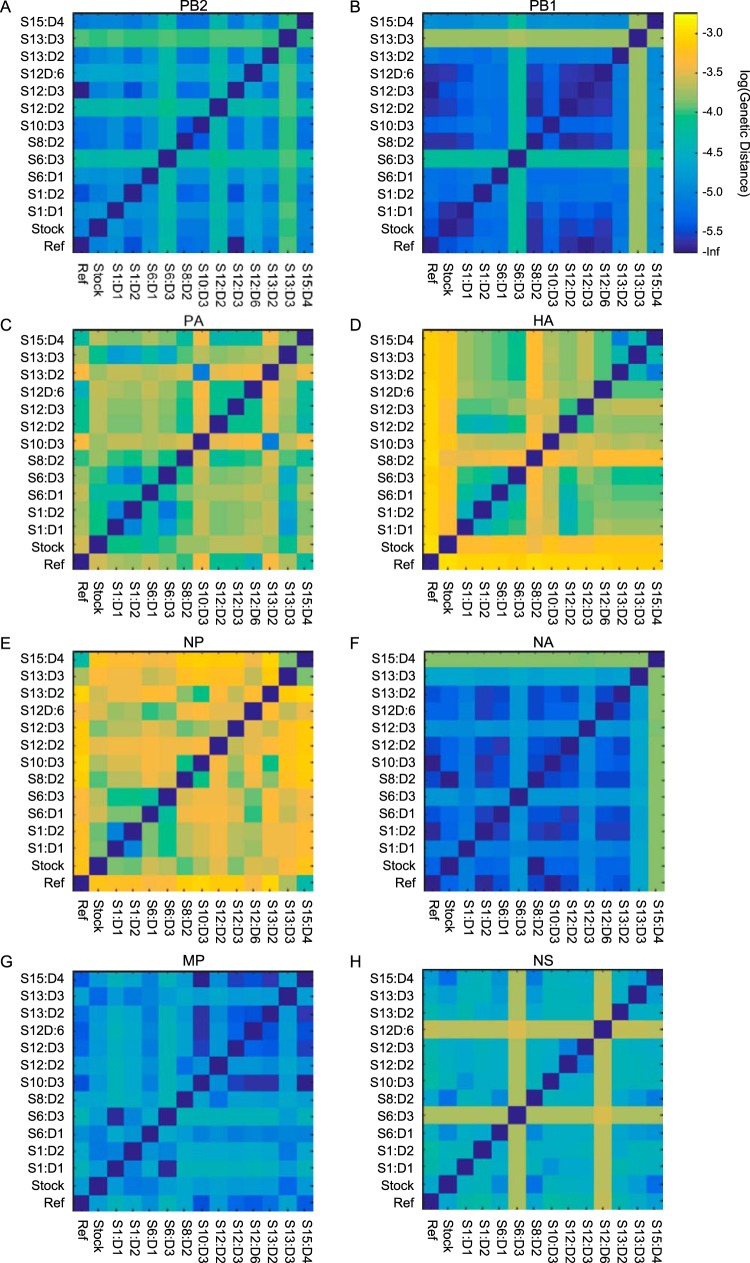
Mean pairwise genetic distance plots between viral populations by gene segment. Ref, reference strain A/Wisconsin/67/2005(H3N2). Genetic distances are shown on the log scale, with lighter colors corresponding to larger mean pairwise genetic distances. Calculation of pairwise distances used sites that had variants called at a 1% cutoff in at least one sample.

As an initial assessment of the extent of IAV evolution that occurred in our study, Fig. 3 shows the pairwise genetic distances between sampled viral populations by gene segment. These distance plots show that the viral populations differed genetically from one another predominantly in 3 of the 8 gene segments: the PA gene segment (which codes for one of the subunits of the RNA polymerase), the hemagglutinin (HA) gene segment, and the nucleoprotein (NP) gene segment. Three key observations can be made from these plots. (i) The large genetic distance between the viral stock and the reference strain [A/Wisconsin/67/2005 (H3N2)], particularly in the PA, HA, and NP gene segments, indicates that viral evolution occurred during generation of the viral stock. (ii) The genetic distances between virus isolated from the subjects' first nasal wash samples and the viral stock can be appreciable, suggesting a narrow transmission bottleneck. Moreover, virus isolated from subjects' first nasal wash samples, particularly for the HA, are often genetically more similar to one another than they are to the viral stock, putatively indicating that the transmission bottleneck is a selective one. (iii) The genetic distances between virus isolated from a single subject at different time points are sometimes substantial, e.g., the distances between S12:D2, S12:D3, and S12:D6. This indicates that intrahost viral evolution occurs throughout the course of infection, either through *de novo* mutation or due to temporal changes in the frequencies of circulating variants. Below, we structure our analyses according to these three observations.

### (i) The viral stock was genetically heterogeneous and partially adapted to the environments experienced during its preparation.

Eight SNPs distributed over 3 gene segments (PA, HA, and NP) were identified in the viral stock (Table 2). This, however, likely represents only a subset of the true variation present within the viral stock, as we were able to characterize only the variants present at ≥1% frequency. Four of these SNPs were nonsynonymous: G70A/D8N, C516A/H156Q, and G1258A/G404R, all on the HA gene segment, and G868A/D290N on the NP gene segment. All of the nonsynonymous variants on the HA segment were present at high frequencies (>10%). The H156Q SNP, located near the receptor-binding domain, has previously been associated with egg adaptation (43). To the best of our knowledge, neither the D8N nor the G404R variant has been associated with egg adaptation in the literature. The G404R SNP was, however, previously identified during passaging of vaccine candidate virus in Vero cells, where it was associated with decreased immunogenicity in ferrets (44). This mutation occurs at the 75th residue of the HA2 peptide, which is located in a region that interacts with HA1 and is known to affect protein stability (44). The D290N SNP on the NP gene segment occurred at low frequency (6%). This variant has been associated with increased disease severity in mice through a replication-independent mechanism (45). The remaining four synonymous variants were identified on the PA and NP gene segments with frequencies ranging from 1 to 57%.

**TABLE 2:**
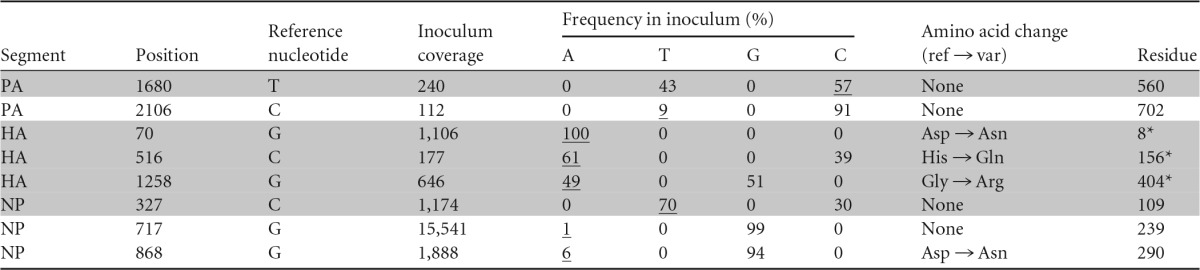
Variants identified in the viral stock^*a*^

a Shaded rows show variants present at frequencies exceeding 10%. Underlined inoculum frequencies correspond to identified variants. *, H3 system numbering (42). All other positions are numbered according to the coding sequence. ref, reference; var, variant.

**FIG 4:**
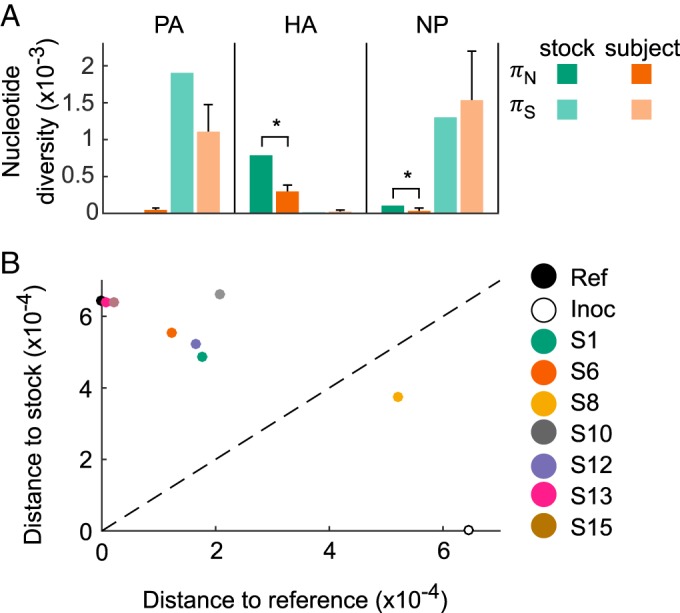
Nucleotide diversity levels and genetic distances. (A) Levels of nonsynonymous and synonymous nucleotide diversity in the viral stock and in the subjects' first nasal wash samples. For subjects, mean diversity levels are shown, with error bars showing standard deviations (SD). In the HA and NP gene segments, π*_N_* is significantly lower in the subjects' first nasal wash samples than in the viral stock (*, *P* < 0.05 using a one-sample Z-test). (B) HA genetic distances between the subjects' first nasal wash samples, the reference strain, and the viral stock, excluding nucleotide site 70. Nasal wash samples are colored by subject. The dashed *x* = *y* line shows where the distance to the viral stock is equal to the distance to the reference strain.

To assess the possible role that selection played on generating observed levels of nonsynonymous genetic variation, we calculated the levels of nonsynonymous nucleotide diversity (π*_N_*) and synonymous nucleotide diversity (π*_S_*) in the viral stock (Fig. 4A). The ratio π*_N_/π_s_* is a commonly used metric for evaluating selection, whereby a π*_N_/π_S_* ratio of >1 is an indicator of positive selection and a π*_N_/π_S_* ratio of <1 is an indicator of purifying selection (46). We found a π*_N_/π_S_* ratio of 143 for the HA gene segment, suggesting that at least one of HA's nonsynonymous SNPs was under strong positive selection during the generation of the viral stock. Due to their high frequencies in the viral stock, their known phenotypes, and the fact that SNP D8N does not contribute to the calculation of π*_N_*, it is therefore likely that either H156Q, G404D, or both rose to high frequency in the viral stock through the process of positive selection in the *in ovo* or the *in vitro* environments, respectively. This conclusion would be consistent with studies that have found that passage of virus in eggs and tissue culture can select for certain amino acids in the HA that are generally not commonly observed in natural influenza virus isolates from humans (43, 44). In contrast to the HA, we found ratios of π*_N_/π_S_* of <1 for both the PA and NP gene segments (π*_N_/π_S_* *=* 5.15 × 10^−4^ and 7.44 × 10^−2^, respectively), signaling purifying selection in these two gene segments.

### (ii) Experimental inoculation was governed by a selective bottleneck.

**FIG 5:**
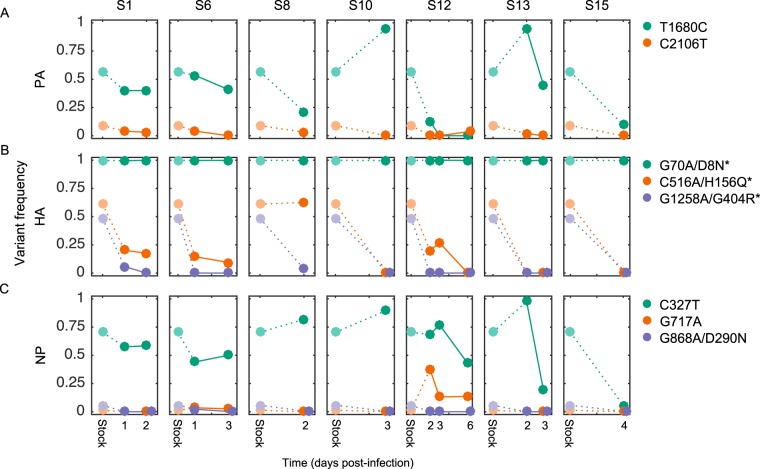
Changes in variant frequencies over time in each of the 7 subjects listed in Table 1. Rows correspond to different gene segments (PA, HA, and NP). Columns correspond to different subjects. Only the dynamics of stock-identified variants are shown. Figure legends list the identities of the plotted variants. *, H3 system numbering.

We next aimed to determine the size and type of transmission bottleneck that occurred following experimental inoculation of the subjects. To this end, we calculated the levels of nonsynonymous and synonymous nucleotide diversity in each of the subjects' first nasal wash samples and compared these levels to those of the viral stock. Figure 4A shows π*_N_* and π*_S_* diversity levels for the PA, HA, and NP gene segments, as these were the only gene segments with variants present in the viral stock (Table 2). In both the HA and NP gene segments, the level of nonsynonymous nucleotide diversity was significantly lower in the nasal wash samples than in the viral stock. In contrast, the level of synonymous nucleotide diversity was not significantly different between the viral stock and the nasal wash samples in any of the three gene segments. These results indicate, first, that the founder viral population size in the subjects was large, as would be expected with direct intranasal inoculation in an experimental infection. The significant reductions in nonsynonymous diversity further indicate that the transmission bottleneck was a selective one, acting to reduce nonsynonymous genetic diversity in the HA and NP gene segments. These reductions can be easily understood in the context of observed variant frequencies in the subjects (Fig. 5). The significant reduction in HA's π*_N_* is due to large decreases in the frequencies of variants H156N and G404R by the time of the subjects' first sequenced nasal wash samples (Fig. 5B). The decreases in these variant frequencies are consistent with both of these variants conferring a fitness cost in human infections, either via a trade-off that facilitates adaptation to a different host environment (H156N, G404R) or by impairment of protein stability (G404R). The significant reduction in NP's π*_N_* is due to observed decreases in the frequency of variant D290N by the time of the subjects' first nasal wash samples (Fig. 5C). It is less clear why NP D290N should confer a fitness cost in human infections, as generally, virulence factors are thought to be associated with increases in viral replication within the host. However, other replication-independent virulence factors that increase virulence while incurring a cost to viral replication have been described for influenza (e.g., PA-X [47, 48]). Thus, it is conceivable that the D290N variant similarly incurs a fitness cost while increasing disease severity.

The presence of a selective bottleneck following inoculation is further confirmed through an analysis of genetic distances between the subjects' first nasal wash samples, the viral stock, and the reference strain, based on the HA gene segment (Fig. 4B). In these calculations, we excluded nucleotide position 70 because the variant at this position (G70A/D8N) went to fixation in the viral stock (Table 2). With the exception of subject S8, all subjects' first nasal wash samples were genetically closer to the viral reference sequence than to the viral stock, indicating that much of the HA genetic variation that originated during generation of the viral stock was purged in the subjects prior to their first successfully sequenced nasal wash sample. This rapid purging of mostly nonsynonymous genetic variation is consistent with a selective bottleneck.

### (iii) Intrahost evolution continues over the course of infection and is dominated by purifying selection.

**FIG 6:**
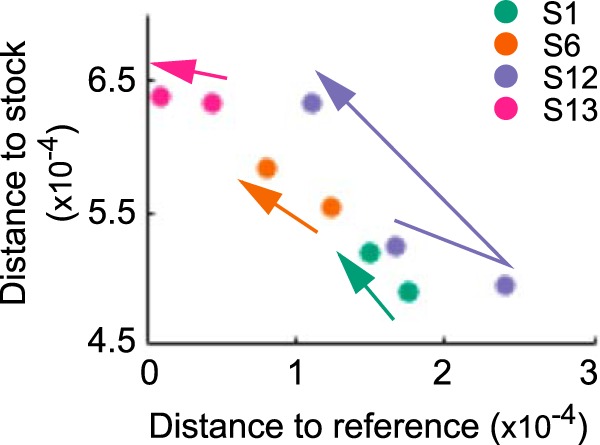
HA genetic distances between nasal wash samples, the viral stock, and the viral reference strain, shown for nasal wash samples of the four subjects with more than one sequenced sample. Arrows between the nasal wash samples show time directionality. As in Fig. 4B, nucleotide site 70 was excluded from the analysis.

To characterize intrahost viral evolution, we first return to Fig. 5, which shows how the frequencies of stock-identified variants change over time in each of the 7 subjects listed in Table 1. A wide range of intrahost frequencies was observed for synonymous variant T1680C on the PA (Fig. 5A). On the HA, the frequencies of variants C516A and G1258A, which were dramatically lower in the subjects' first nasal wash samples than in the viral stock, tended to decrease further over the course of infection in the four subjects with more than one sequenced nasal wash sample (Fig. 5B). Nonsynonymous HA variant G70A, which went to fixation in the viral stock, remained fixed in all subjects over the course of infection. On the NP, a wide range of intrahost frequencies was observed for synonymous variant C327T, with no consistent directional pattern that was apparent (Fig. 5C). Similar to the dynamics of nonsynonymous variants on the HA, the frequency of nonsynonymous variant G868A on the NP, which was lower in the subjects' first nasal wash samples than in the viral stock, tended to decrease further over the course of infection in the four subjects with more than one sequenced nasal wash sample (Fig. 5C). Overall, the variant frequency dynamics shown in Fig. 5 point toward continued purifying selection acting on the variants that appear to be responsible for the selective bottleneck. To further support the conclusion of continued purifying selection on the HA, Fig. 6 shows how genetic distances between the viral reference strain, the viral stock, and the nasal wash samples change over time, using data from the four subjects having more than one sequenced nasal wash sample. With the exception of subject S12, nasal wash samples progressively become genetically closer to the reference strain over time. Note that nasal wash sample S12:D3 has exceptionally low coverage for the HA gene segment (Fig. 2), limiting our interpretation of the dynamics in this subject. In addition, as noted above, all subjects were treated with oseltamivir on day 5 postchallenge, and therefore, we cannot exclude the possibility that this treatment affected the nucleotide diversity and variant composition in nasal wash sample S12:D6.

In addition to frequency changes of variants transferred during experimental infection, intrahost viral evolution may also proceed through the emergence of *de novo* mutations. To examine the importance of *de novo* mutations in contributing to intrahost viral genetic diversity in the challenged subjects, we identified variants in the subjects' nasal wash samples that were not present in the viral stock. Note that these variants may have originated in the viral stock and been transferred to the subjects, rather than being bona fide *de novo* mutations. Unfortunately, with the coverage obtained in this study, it is not possible to distinguish between the case of *de novo* mutation and the case of inoculation with a very low frequency variant. More accurate, ultrasensitive sequencing methods, such as those described in reference 49, would need to be used to truly distinguish *de novo* variants from low-frequency minority variants present at the onset of infection (50). The observation that 97% of the putative *de novo* variants were identified in a single nasal wash sample favors the possibility that at least some of these variants likely evolved *de novo* in the subjects.

**FIG 7:**
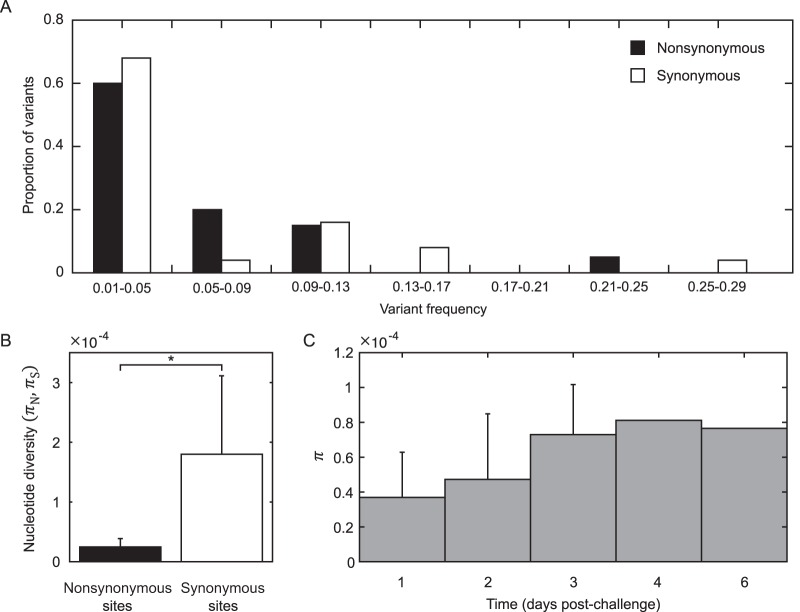
Putative *de novo* variants identified in challenge study subjects. (A) Nonsynonymous and synonymous variant frequencies. (B) Nonsynonymous and synonymous nucleotide diversity, calculated from the subjects' nasal wash samples using only *de novo* variant SNP sites. π*_N_* was significantly less than π*_S_* (*, *P* < 0.01, using the paired *t* test). (C) Total nucleotide diversity (π) as a function of time since inoculation. Error bars show SD for time points that included >1 sample. Only *de novo* variant SNP sites were included in the analysis.

Twenty of the 44 identified variants were nonsynonymous variants; the remaining 24 were synonymous variants. None of these variants has previously been characterized in the literature. The frequencies of identified nonsynonymous variants did not differ significantly from the frequencies of identified synonymous variants (Fig. 7A). The level of nonsynonymous nucleotide diversity contributed by these putative *de novo* variants, however, was significantly lower than the level of synonymous nucleotide diversity (Fig. 7B). These findings indicate that purifying selection is a major factor limiting nonsynonymous genetic variation in this study. Occurring at frequencies similar to those of synonymous variants, the observed nonsynonymous variants were likely either neutral or nearly neutral. Calculations of overall nucleotide diversity, plotted by day postinoculation, indicate that IAV genetic diversity increases only slightly over time, with the increase not being statistically significant (Fig. 7C). Thus, while it appears that *de novo* variants can evolve during acute infection of otherwise healthy human hosts, they do not lead to rapid intrahost genetic diversification and their dynamics are overshadowed in this study by the dynamics of the genetic variants that arose during generation of the viral stock.

## DISCUSSION

We have here analyzed viral deep sequencing data from nasal wash samples of 7 subjects challenged with human IAV. The viral stock used for inoculating the subjects was generated by propagating reference strain A/Wisconsin/67/2005 (H3N2) sequentially through avian primary chicken kidney cells, embryonated chicken eggs, and GMP Vero cells. Based on the nonsynonymous mutations that were identified in the viral stock (Table 2) and on the levels of nonsynonymous (relative to synonymous) nucleotide diversity present in the stock (Fig. 4A), it appears that the virus stock was at least in part adapted to the egg and/or tissue environments that it experienced during its preparation. The significant reductions in nonsynonymous nucleotide diversity (π*_N_*) on the HA and NP gene segments, in the absence of significant reductions in synonymous nucleotide diversity (π*_S_*) on any gene segment, are consistent with a selective transmission bottleneck following inoculation (Fig. 4A). This conclusion was strengthened by the finding that the subjects' first sequenced nasal wash samples were genetically more similar to the reference strain than to the viral stock (Fig. 4B). Over the course of infection, the intrahost viral population continued to undergo purifying selection (Fig. 5), with a tendency to become genetically even closer to the reference strain (Fig. 6). Although a large number of nonstock variants were identified in the nasal wash samples (Fig. 7A), these putative *de novo* variants did not appear to be subject to strong positive selection and their appearance did not result in rapid intrahost genetic diversification (Fig. 7C).

Our observation of apparent adaptation during the preparation of the viral stock is consistent with previous studies showing that certain nucleotide substitutions lead to improved replication *in ovo* (51–54) or *in vitro* (44, 55). While these variants can be beneficial in some circumstances, as in the production of vaccines by increasing the yield of virus (51, 54), they may also have unforeseen effects on viral antigenicity and immunogenicity and thus on vaccine efficacy (56, 57). In fact, the H156Q variant that was enriched in the viral stock of this experiment affects a major antigenic site (58) and was demonstrated to have contributed to the decreased vaccine efficacy during the 2012-2013 influenza season (43).

Our finding of a strong selective bottleneck acting on the HA and NP gene segments is consistent with gain-of-function experimental studies that have characterized the transmission bottleneck of avian-adapted IAV between index and contact ferrets (18, 19). The selective bottleneck observed in these transmissions led to a sharp reduction of nonsynonymous genetic diversity on the HA gene segment, while genetic diversity on the other gene segments remained intact. In our study, selective forces appeared to act not only on the HA gene segment, but also on the nonsynonymous genetic variation present on the NP gene segment.

Our study further found that intrahost evolution was dominated by persistent purifying selection, with only limited genetic diversification arising via putative *de novo* mutation. This stands in contrast to the experimental gain-of-function studies (17, 18), in which rapid genetic diversification of the IAV population occurred following transmission of only a small number of haplotypes. Our study is more consistent in this manner with intrahost evolutionary dynamics observed in studies in which the virus is already well adapted to its host (9, 10, 13). Yet, in contrast with our current findings, these studies found transmission bottlenecks to be loose and nonselective.

**FIG 8:**
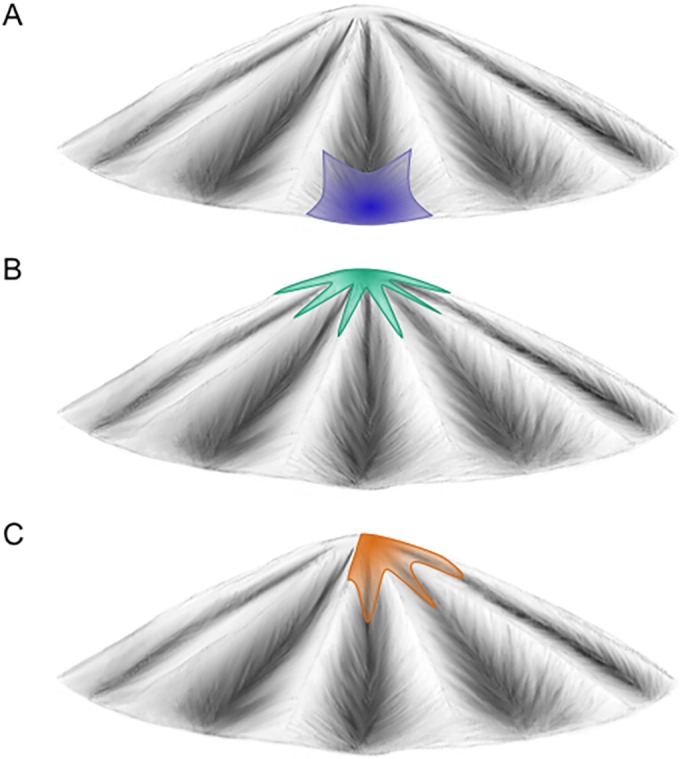
Occupation of an idealized fitness landscape by a virus poorly adapted to its host (A), a virus well adapted to its host (B), and the viral stock described in this study within a human host (C).

To reconcile the differences between the findings of our study and those from these previous studies, in which the virus is either poorly or well adapted to its host, we can consider an idealized fitness landscape occupied by a viral population (Fig. 8). In the case of a virus that is poorly adapted to its host, such as an avian IAV in a ferret, the initial viral population occupies an area around a fitness valley (Fig. 8A), with some virions having higher fitness than others in the novel host environment. Seeding of this viral population into a contact animal results in a selective bottleneck, with a small number of virions having a major selective advantage over other virions. Once this small number of virions has established, the viral population has many further mutational routes available to it to further increase fitness. This leads to rapid intrahost genetic diversification. In contrast, for a well-adapted virus, the initial viral population already occupies a fitness peak (Fig. 8B). Because there is very little difference in the fitness levels of virions within the population, transmission is characterized by a loose, nonselective bottleneck. Within newly infected hosts, the virus already occupies a fitness plateau, such that most mutations are either deleterious or neutral. As a result, little intrahost viral genetic diversification occurs, and evolution will be dominated by purifying selection. In our study, the virus is generally well adapted to humans but contains variants that have been selected for during stock generation. While these variants likely had a fitness advantage during virus propagation, these variants likely carried a fitness cost in human hosts. As a result, the initial viral population can be thought of as occupying a region of the fitness landscape that spans the peak, as well as several nearby gulches (Fig. 8C). Because of the fitness differential present in the viral population, this fitness landscape occupancy leads to a selective transmission bottleneck and continuing intrahost purifying selection. Little intrahost genetic diversification is apparent, because most mutations are deleterious or, at best, neutral.

While a selective bottleneck and limited viral genetic diversification may—in hindsight—not be surprising, a notable result is the speed at which intrahost viral adaptation can occur. By the first successfully sequenced nasal wash sample, the nonsynonymous variants that were enriched (but not fixed) *in ovo* or *in vitro* had either markedly decreased in frequency or were absent entirely from the sample. This suggests that selection pressures can be strong in healthy and effectively naive human hosts and that high levels of genetic diversity at the onset of infection enable the occurrence of rapid intrahost evolution.

One major limitation of this study was the short read lengths of the viral sequences, which curtailed our ability to distinguish those sites under direct selection from those loci whose allele frequency changes could have been driven by linkage to other alleles. Given that homologous recombination does not readily occur in influenza viruses (59), the contribution of linkage must be considered when interpreting the frequency changes of variants on the same gene segment. Given that only one high-frequency nonsynonymous variant was identified on the NP in the viral stock, however, it is likely that the reduction in nonsynonymous genetic diversity on the NP gene segment following subject inoculation was a consequence of selection directly acting against this G868A variant. On the HA gene segment, it is less clear whether selection acted against C516A, G1258A, or both. Longer reads would prove invaluable in determining which HA sites were under selection and which variants changed in frequency simply as a result of genetic hitchhiking. Longer reads would also allow us to identify epistatic interactions between sites, similar to what was done in recent work based on IAV deep sequencing data from an H5N1 host adaptation study (60).

Despite this limitation, we have shown that an IAV population partially adapted to the environments in which it was generated can rapidly evolve in healthy and effectively naive human hosts. This rapid evolution occurs via a selective transmission bottleneck and continuing purifying selection acting on the founding viral population. While a large and phenotypically heterogeneous founding population size may be unique to this experimental study, recent estimates of transmission bottleneck sizes in natural human-to-human infections are much larger than anticipated by many—on the order of 100 to 250 virions (16). This indicates that the fitness differences within a viral population, rather than the initial levels of genetic diversity, may be the primary factor setting the speed limit of IAV adaptation. Clearly, further analyses are critical for improving our nascent understanding of the dynamics of IAV adaptive evolution between transmission events and within single infections. This work, however, provides support to existing studies documenting the remarkable capacity for this virus to rapidly evolve, this time in human hosts.
